# Blood flow mechanics in cardiovascular development

**DOI:** 10.1007/s00018-015-1885-3

**Published:** 2015-03-24

**Authors:** Francesco Boselli, Jonathan B. Freund, Julien Vermot

**Affiliations:** 1 Institut de Génétique et de Biologie Moléculaire et Cellulaire, Illkirch, France; 2 Centre National de la Recherche Scientifique, UMR7104, Illkirch, France; 3 Institut National de la Santé et de la Recherche Médicale, U964, Illkirch, France; 4 Université de Strasbourg, Illkirch, France; 5 Mechanical Science and Engineering, University of Illinois at Urbana-Champaign, Urbana, IL 61801 USA

**Keywords:** Angiogenesis, Fluid mechanics, Valvulopathie, Atherosclerosis, Klf2, Biomechanics, Cardiomyopathy

## Abstract

Hemodynamic forces are fundamental to development. Indeed, much of cardiovascular morphogenesis reflects a two-way interaction between mechanical forces and the gene network activated in endothelial cells via mechanotransduction feedback loops. As these interactions are becoming better understood in different model organisms, it is possible to identify common mechanogenetic rules, which are strikingly conserved and shared in many tissues and species. Here, we discuss recent findings showing how hemodynamic forces potentially modulate cardiovascular development as well as the underlying fluid and tissue mechanics, with special attention given to the flow characteristics that are unique to the small scales of embryos.

## Introduction

The cardiovascular network is constantly subjected to the mechanical forces generated by the beating heart. The heart beat starts early in embryonic development, so cardiovascular development is dynamic: endothelial cells reorganize and migrate to form an efficient vascular network at the same time that blood flow increases with the increasing efficacy of the beating heart. From a developmental biology standpoint, this suggests evolving interactions between hemodynamic forces, heart function, and cardiovascular morphogenesis. These interactions are increasingly being recognized as important as the influences of mechanical forces become better understood. Embryonic hemodynamics are characterized by unique flow regimes and necessitate the introduction of fluid mechanics concepts into our understanding of cardiovascular development. The key elements associated with hemodynamic forces, such as the flow-sensing mechanism and flow responsive genes, are now beginning to be identified and quantified. This provides a quantitative understanding of the sensitivity and the range of response of endothelial cells to flow forces during development. More generally, many of these advances have benefited from the use of animal models that have provided unprecedented experimental approaches, particularly live imaging. For example, advances based upon studies of tissue morphogenesis in *Dosophila* *melanogaster*, *c. elegant*, zebrafish, and chicken have demonstrated how mechanical forces couple with development and provide a framework for understanding development at the cellular scale [28]. Enabled by these models and technologies, new concepts integrating gene networks, cell mechanics, and mechanical forces are currently emerging. Particular progress has been made in understanding cardiovascular development [21], in particular that of heart valves [64, 71], trabeculae [68], and blood vessels [23, 29]. In this review, we discuss recent advances in cardiovascular biomechanics and its role in cardiovascular development, with an emphasis on the potential impact of the different feedback loops between forces and genetics recently uncovered in the process. Throughout the review, we explain why flow mechanics itself represents an essential aspect of this coupling.

## Pumping mechanisms and flow functions in the developing heart

Studies on blood flow dynamics in the developing heart are in constant progress. Both the heart contraction and blood flow have an impact on the cellular organization that leads to heart morphogenesis. Here we discuss how flow forces change as the heart develops and describe both the heart biomechanics during development and how the resulting forces impact in controlling cardiac development. The conservation of certain mechanisms discussed has yet not been confirmed across different animals; when this is the case, the animal model will be specified.

### Early heart development: valveless tube to chambered heart to heart valve formation

**Fig. 1:**
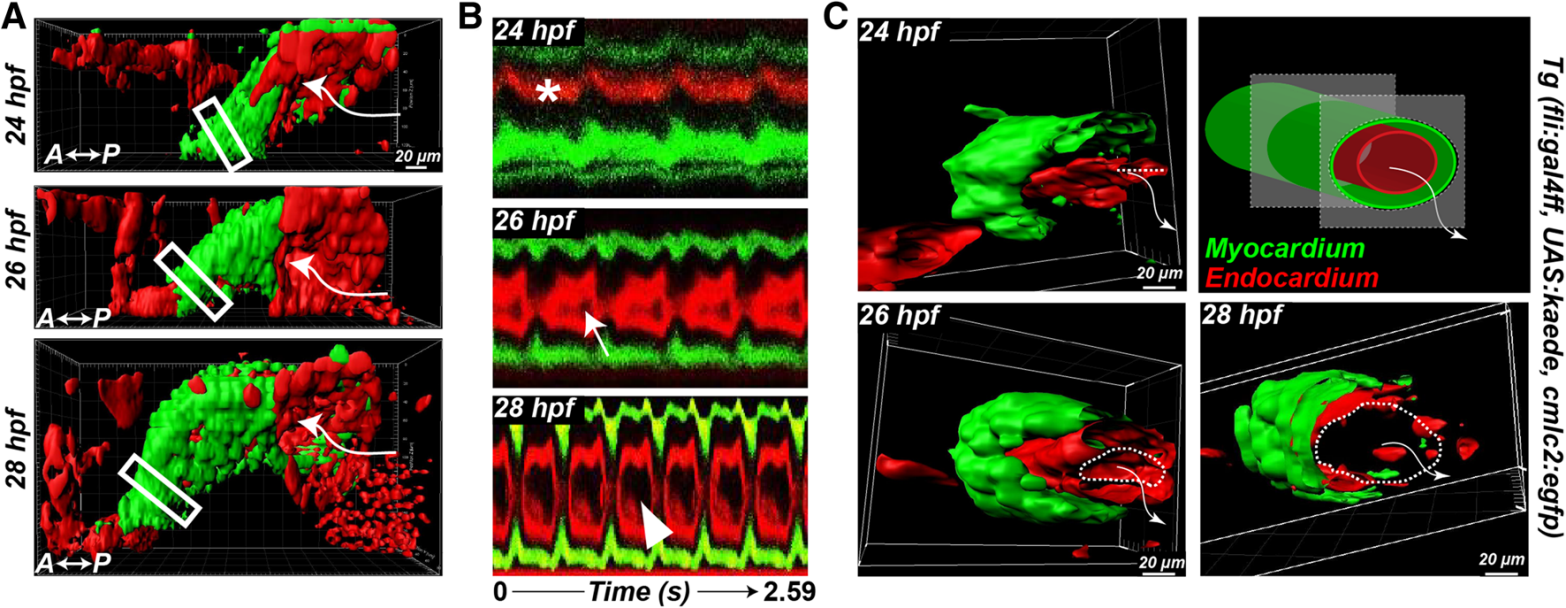
Onset of embryonic heart contractions. **a** 3D images of the heart beat in a Tg (cmlc2:egfp; fli:gal4FF; UAS:kaede) embryo. The myocardium is labeled with *green* (GFP); the endocardium and blood cells are *red*. **b** Kymographs of a section (*boxed*) of the heart tube. The *asterisk* indicates the absence of a visible endocardial lumen at 24 hpf. **c** 3D sections of the *boxed regions* in (**a**). The *dashed lines* indicate the endocardium opening; the *arrows* show the flow direction. Adapted with permission from Goetz et al. [25]

*Initiation of heart contraction*   At the onset of blood circulation, the heart is a tubular structure with a wall made up of three layers (Fig. 1): an outer layer of contractile myocytes and an inner layer of endocardial cells in contact with the blood are separated by an elastic, cell-free layer known as the cardiac jelly.

In zebrafish, myocardial contractions start about 24 hpf, when the heart is incompletely lumenized and the flow is very low. Shortly thereafter, the lumen starts opening and the stroke volume and the frequency quickly increase. About 28 hpf, the heart beats regularly at 2.6 Hz. A contraction wave starts at the inflow and propagates along the entire length of the heart tube with uniform speed. The contraction wave squeezes different cross sections of the tube sequentially (Fig. 1), pushing blood into the aortic arches and the dorsal aorta (DA) without significant backflow despite the lack of valves at this stage. This contraction is significantly shorter than the entire heart cycle (about 50 %) [35], which is reflected by the generation of pulsatile flow (pulsatility index $(U_{\mathrm{max}}-U_{\mathrm{min}})/U_{\mathrm{mean}} = 2$) [25], regardless of the contraction wave speed. Thus, at this stage, the heart tube is a valveless pulsatile pump. The myocardial depolarization signal resembles the contraction pattern and also propagates longitudinally from the inflow tract to the outflow tract [14]. Interestingly, it is the lumen diameter and not the heart rate that best correlates with the flow rate at these early stages [25].

**Fig. 2:**
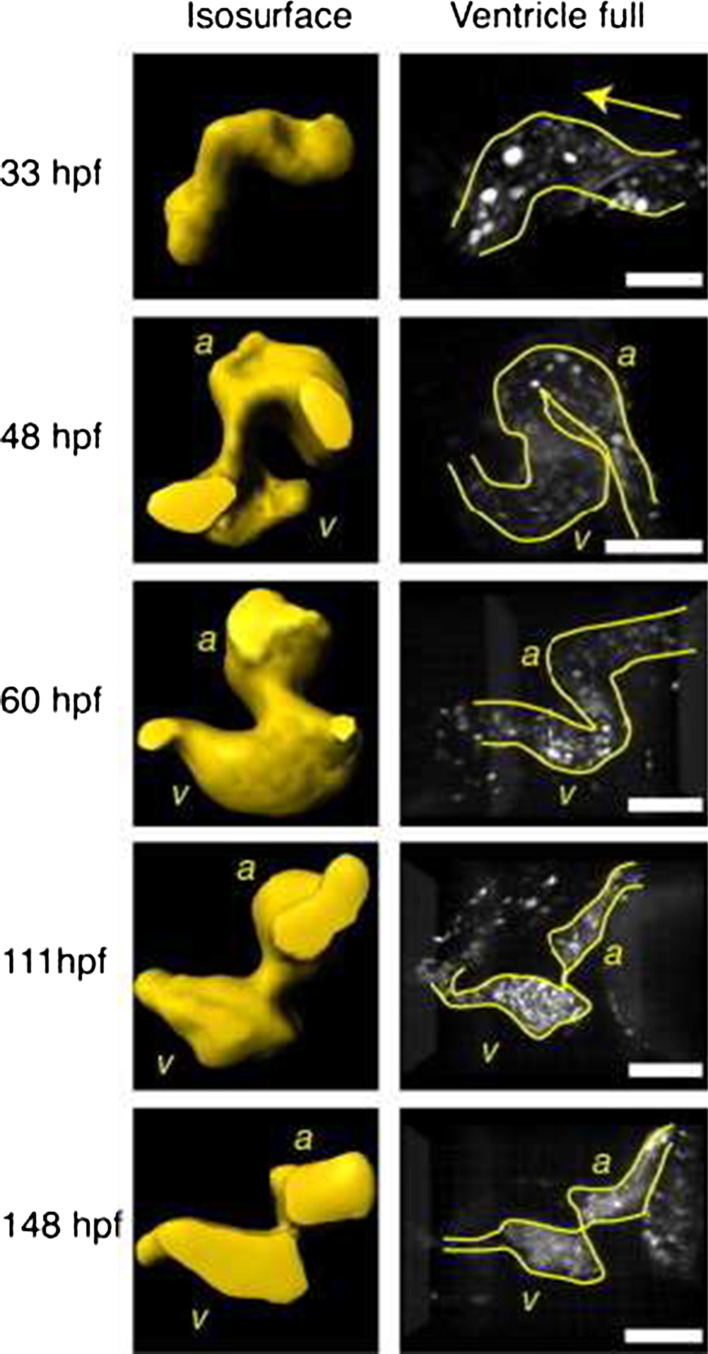
Developing Tg (gata1:GFP) zebrafish heart at various stages. *Left column* isosurface renderings obtained at mid-diastole. *Right column* volume renderings (maximum intensity projection) when the ventricle is full. *a* atrium; *v* ventricle. *Arrows* indicate flow direction. *Scale bars* 100 μm. Adapted with permission from Liebling et al. [41]

*Early looping*   Soon, the heart undergoes a conformational change known as looping, and the ventricle and the atrium expand (ballooning). At the same time, the constricted region between the forming atrium and the ventricle specializes, becoming the atrio-ventricular canal (AVC), where the atrio-ventricular valve will form (Fig. 2).

**Fig. 3:**
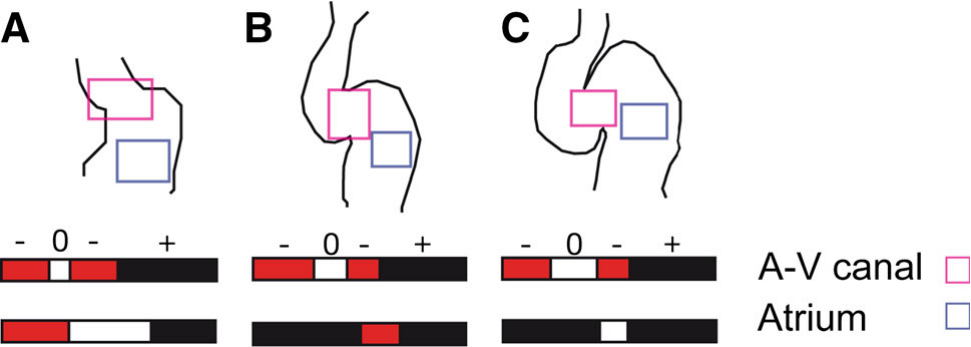
Average transvalvular flow direction as a function of time for wild-type hearts as seen in the AV canal (*light magenta box*) or atrium (*light blue box*) between **a** 36 hpf, **b** 48 hpf, and **c** 58 hpf in the area highlighted in the *heart drawings*. Anterograde flow from the atrium to ventricle is shown in *black*, retrograde flow from the ventricle to the atrium in *red*, and no flow between the chambers is shown in *white*. The sequence of time segments with retrograde, anterograde, and no-flow fractional periods is depicted in *red*, *black*, and *white*, respectively. Adapted with permission from Vermot et al. [71]

The contraction pattern of the atrium is similar to that of the heart-tube stage, but the contraction now significantly slows along the AVC and the ventricle, such that it makes up almost the entire heart cycle, with the next contraction wave starting almost immediately after the end of the previous wave. Although the flow is unidirectional in the vasculature, the contraction wave pattern and timing, as well as the geometrical variation, produce a significantly oscillatory flow in the zebrafish heart. A significant fraction of blood volume is pushed back into the sinus venosus upstream of the heart by the atrial contraction and into the atrium by the ventricular contraction (Fig. 3). Since there is dissipation associated with such flow reversal (or regurgitation), from a technical point of view, this makes the heart energetically inefficient. Yet, these reversing flows are biologically important, because they expose the endocardial cells of these regions to locally distinct hemodynamic stresses. Furthermore, this occurs at a stage when the heart undergoes fast and flow-dependent morphogenetic changes. Indeed, these factors are related: blood flow is an essential epigenetic factor for chamber ballooning, conduction system formation, and valve formation [5, 14, 17, 32, 71].

*Late looping and cushion formation*   As looping progresses, the ventricle repositions next to the atrium, and the endocardium at the AVC thickens to form valve precursors known as cushions. At 48 hpf, the side of the wall of the AVC where the curvature is higher is obviously thicker in the zebrafish [64]. The atrium and ventricle are now clearly defined (Fig. 2) [41]. The contraction pattern of the atrium has not changed significantly: a contraction wave still propagates from the inflow region of the atrium to the AVC. The contraction wave still slows significantly when it reaches the AVC such that atrial and ventricular systole appear now as two successive and distinct processes. The time needed by the contraction wave to travel along the heart is now slightly longer than the heart cycle: the atrium restarts contraction slightly before the end of ventricular contraction [12, 35].

**Fig. 4:**
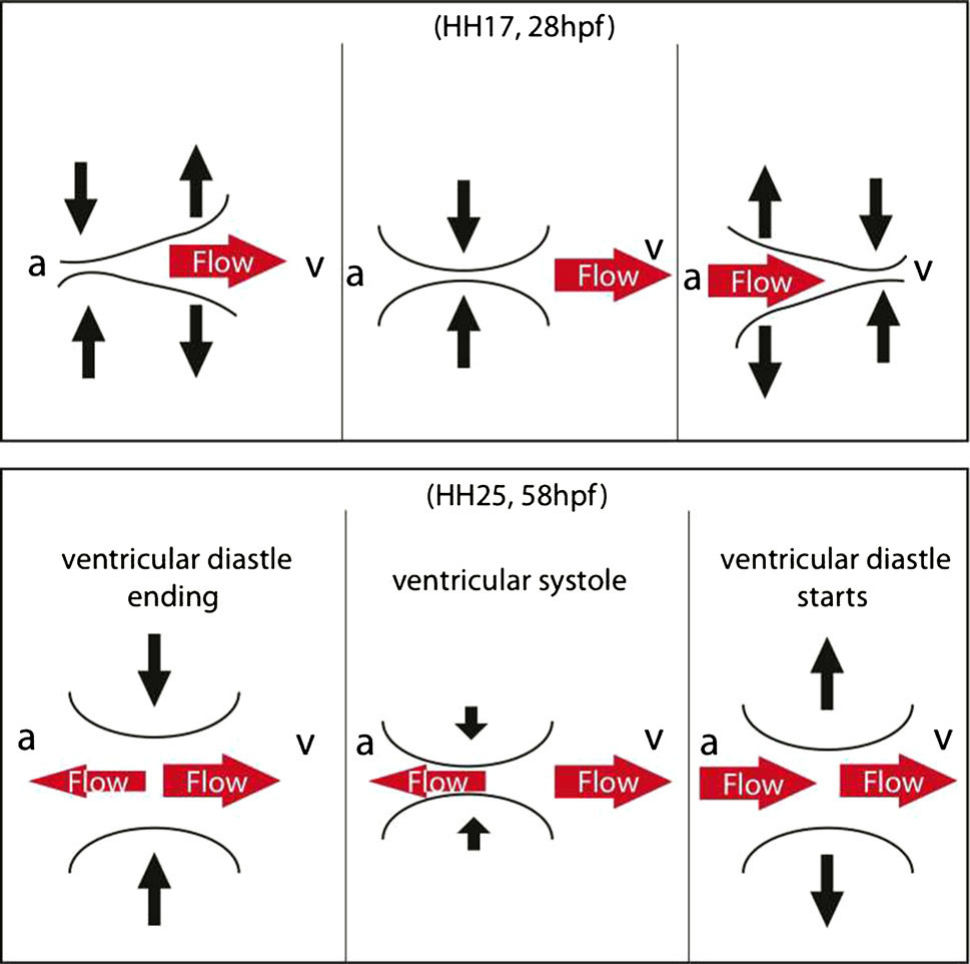
Developmental transition of cushion dynamics observed in chicken from HH17 to HH25 [12]. The concomitant transition of flow in and nearby the AVC cross section is illustrated by the *red arrows* as observed in zebrafish around 28 and 58 hpf [25, 64, 71]. *Arrows* pointing to the *left* represent possible reversing flow from ventricle (v) to atrium (a)

In contrast to the atrium, the ventricle now contracts more uniformly, as in the adult heart. Most interestingly, the AVC contracts during most of the ventricular systole [35], which together with the growing endocardial cushions resists back flow from the ventricle and thereby serves a valve-like function. The atrium lacks inflow cushions yet still functions as a valveless pump, though with more complex flow dynamics since the geometry deviates significantly from the initial simple tube. In contrast to the mature valves, which passively close to prevent any significant back flow, the cushions require a complex synchronization of the active contractions in the atrium, the AVC, and the ventricle to ensure unidirectional flow. Even so, there remains significant reversing flow in the AVC (Figs. 3, 4), which mediates the further development of endocardial cushions into mature valves [71]. Reversing flows have been shown to induce the expression of the flow responsive gene klf2a. This step is essential for the subsequent heart valve morphogenesis [71].

*AVC cushions: development and dynamics*   The development of the cushion is associated with changes in their mechanical properties, their dynamics, and their effectiveness in performing the role of unidirectional valves. The early cushions are acellular, starting as thicker region of the jelly layer. In the chicken embryo, this stage is observed at HH17 (Hamburger Hamilton Stage 17) [27]. They thicken dynamically in phase with the myocardium, with minimum and maximum thicknesses during filling and peak myocardial contraction, respectively. As they develop, the AVC cross section becomes transiently closed for a small period of the heart cycle, while blood flows through the AVC most of the time (Fig. 4).

Butcher et al. [12] observed two flow phases at this stage in the chicken AVC: one during the filling, which corresponds to the elastic restoration of the cardiac wall, and one during myocardial contraction. During this same stage, the mesenchymal cells that will form the valve produce collagen fibers and remodel the extracellular matrix in a fashion that thicken the cushions [12]. At HH25 the cushions start to cellularize, they appear relatively rigid, and their thickness ceases to change significantly. Under these conditions, cushions move in an unbending apparently uniform motion rather than a propagating contraction as at HH17 (Fig. 4). The AVC cross section is now open for only about one-third of the heart cycle (and the flow velocity now has a single peak rather than a biphasic time profile). Butcher et al. [12] identified an intriguing transitional condition at HH21, when the thickening of the cushions becomes out of phase with the myocardial contraction with the cushions assuming their minimum thickness about 0.2 cycle (70°) after myocardial contraction starts. It is tempting to associate this with the relaxation time constant of the cushions, which is expected to be inversely proportional to their elasticity.

*Cardiac conduction system and the emergence of AVC conduction delay*   Cardiomyocytes form the cardiac conduction system which coordinates chamber contraction and drives blood pumping. The changes in the contraction pattern reflect the evolution of the calcium depolarization wave in the myocardium which is strongly flow and contractility dependent [14, 15]. Initially, the depolarization starts at the superior part of the atrium, and propagates linearly along the heart tube. However, this transitions into a binodal configuration shortly thereafter. In chicken, a second wave is initiated in the ventricle at HH25, which is observed following atrial depolarization [12].

**Fig. 5:**
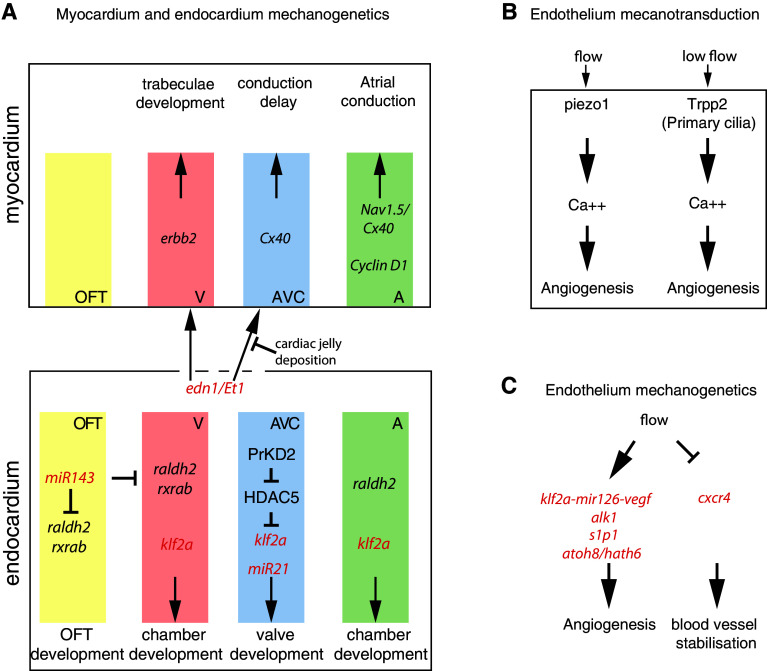
Schematic of the mechanotransduction signaling pathways in the developing heart. **a** Flow-dependent cardiac genetic pathways leading to outflow tract (OFT), heart chambers, and valve formation. Myocardium and endocardium specific genes are grouped into the *top* and the *bottom panel*, respectively, and *colors* point out their specificity to the different parts of the heart: OFT, ventricle (V), atrium (A), and atrio-ventricular canal (AVC). **b** Endothelial, blood flow mechanosensors, and **c** flow responsive genes involved in angiogenesis. **a**–**c** Flow responsive genes are in *red*

The desynchronization between atrial and ventricular contraction is mediated by AVC conduction delay, i.e., calcium waves get slowed down in the AVC. Interestingly, the AVC conduction delay is locally defined by the morphogenetic events that initiate valve formation. Bressan et al. [8] recently showed that the physical separation from endocardial-derived factors prevents the AVC myocardium from activating fast conduction markers (*Cx40*) and becoming fast conducting (Fig. 5a). Mechanistically, this physical separation is induced by cardiac jelly deposition by the myocardial cells at the onset of valve formation. The conduction patterning occurs via reciprocal myocardial-endocardial interactions and valve formation is coordinated with establishment of a conduction delay. Overall, this work exposes another type of biomechanical coupling between heart function and morphogenesis. Importantly, such a mechanism can lead to synchronization of the electrophysiological and structural events necessary for the optimization of blood flow through the developing heart [8]. It is interesting to note that endothelin1 (*edn1/Et1*) is secreted by the endocardial cells to regulate this process. As blood flow controls endothelin1 expression [26], it is likely that the reciprocal interactions between the AVC conduction delay and the endocardium is mediated by flow (Fig. 5a). Moreover, hemodynamic factors regulate the development of the atrial conduction system in chicken. Here, the stretch experienced by atrial cardiomyocytes due to hemodynamic stresses triggers proliferation and the expression of proliferation (*Cyclin D1*) and fast conduction (*Cx40, Nav1.5*) markers, which in turn promotes the formation of the large diameter muscle bundles necessary for efficient conduction [9] (Fig. 5a).

*Outflow tract development*   The dynamics of the outflow tract (OFT) was only recently characterized in the chicken embryo at HH18 [43]. The OFT shares a similar myocardial-cardiac jelly-encodardium structure as the heart, and myocardial contractions are observed at the interface with the ventricle. This leads to a contraction wave that propagates from the ventricle into the aorta, which causes complex dynamics of the jelly layer. Overall, the OFT is occluded during ventricular diastole and thus suppresses backflow. We are not aware of further studies of the OFT at later stages. The outflow tract was also suggested to act as a capacitor that maintains continuous flow in the branchial arches [33]. It is of note that endocardial cells express high levels of klf2 in this area [26, 71] suggesting that flow might be involved in its morphogenesis as well. In zebrafish, flow is necessary to activate the expression of *miR-143* in order to suppress retinoic acid signaling (*raldh2, rxrab*) in the OFT. The heartbeat regulates cardiogenesis by suppressing retinoic acid signaling via *miR-143* expression (Fig. 5a) [49].

*Endocardial chamber morphogenesis*   During heart development, the onset of the heartbeat and blood flow coincides with a ballooning of the cardiac chambers. The endocardial cells obtain distinct chamber-specific and inner- versus outer-curvature-specific surface areas in response to flow. Recent work in zebrafish showed that the hemodynamic-sensitive transcription factor klf2a is involved in regulating endocardial cell morphology. These findings demonstrate that the endocardium is a flow-sensitive tissue and suggest that endocardial cells can adapt chamber growth in response to blood flow [17].

*Valves maturation*   In zebrafish, the transformation of cardiac cushions into valve leaflets happens between 48 and 96 hpf [6, 41] and by 111 hpf the valves are functional and unidirectional flow is established at both the AVC and the outflow tract [41, 64]. At 148 hpf, the ventricular filling starts having a biphasic character: a passive filling phase begins before the atrium contracts followed by an active diastolic phase that is driven by atrial contraction. This behavior is similar to that of the adult human ventricle.

*Trabeculation*   Concomitantly with the development of cardiac cushions and the end of cardiac looping, portions of the myocardium project from the ventricular outer curvature into the lumen to generate the trabeculae [24, 68]. Around 3 dpf in zebrafish, trabeculae appear as circumferential ridges [57], that later replace the cardiac jelly by a complex trabecular network, a spongy layer surrounded by a thin and compact myocardial layer [57, 69]. At 5 dpf, the zebrafish ventricle develops extensive trabeculation, but the inner surface of the atrium remains smooth [57]. In other animals, this process seems to start earlier, e.g., in chicken at HH17, when the ventricle still works in a “peristaltic” fashion [65]. After septation of the chicken heart, about HH34, the trabeculae reorganize from a radial to an apico-basal orientation [65]. Later, a process known as trabecular compaction starts that increases the thickness of the compact myocardial layer [33, 65].

Trabeculation is considered a fundamental developmental step toward proper heart function, though little is known about the function of trabeculae. It has been suggested that trabeculae may impact both electrical and mechanical properties of the heart. Trabeculation increases endocardial mass corresponding to the growing load of the developing vascular system. At the same time, trabeculae significantly increase the endocardial surface, which may facilitate oxygen supply in the absence of the coronary arteries that will form only later [65, 66]. Although seemingly necessary for the development of a healthy mature heart, the role of trabeculae on the mechanical pumping mechanisms is not yet resolved. Several studies suggest they contribute to the conduction system of the embryonic ventricle [66] and therefore on the dynamics of myocardial depolarization. From a mechanical point of view, comparison between trabeculated and smooth models of the adult ventricle suggest that trabeculae increase the compliance of the ventricle, favor ventricular filling, and increase the stroke volume [67]. These results significantly depend on the orientation of the trabeculae. Their actual role may evolve over time during their reorganization. Moreover, mechanical shear stresses are higher at the trabeculae [10, 67], which was suggested to guide mechanotransduction and morphogenesis and therefore the development of the heart pump [10].

The genetics associated with trabeculae formation in response to flow starts to be uncovered. Endothelin 1 (*edn1/Et1*) is expressed in the endocardium and activates *erbb2* signaling in order to activate trabeculae formation, by modulating myocardial cell proliferation [44] and the myocardial cell protrusive activity necessary for trabeculae formation [68] (Fig. 5a).

*Epicardium morphogenesis*   The epicardium corresponds to the outer cell layer that is in contact with the myocardium and forms once the heart has looped. It plays an important trophic role during cardiac development by nourishing the myocardium and generating the progenitors that give rise to intracardiac fibroblasts and the coronary vasculature. In zebrafish, the epicardium originates from clusters of cells that are located in the pericardial cavity outside of the heart. The formation of the cluster and the colonization of the myocardium by the epicardial progenitors is strikingly dependent on the heart beat. As a consequence of the heartbeat, fluid advections are generated outside the heart. A recent study showed that the pericardial flow streamlines are very efficient in propelling the precursors of the epicardial cell layer toward the myocardial cell surface [56]. Overall, the heart beat generates many types of flow leading to several morphogenetic processes: intracardiac flows that are necessary for trabeculation, endocardial chamber and valve development, and extra cardiac flows that are necessary for epicardium development.

### Early heart beat: mechanotransduction and mechanogenetics

The mechanotransduction operating in the developing heart is not well established. However, a number of signaling pathways and tissue interactions involving mechanotransduction have been described (Fig. 5a). The best known flow responsive genetic pathway in the growing heart corresponds to the one activated in the AVC to control valve morphogenesis in zebrafish. It followed from the discovery that the transcription factor *klf2a* is activated in response to reversing flow in the AVC and is necessary for valve development. More recently, studies have then demonstrated that * klf2a* is under the control of PrKD2 (protein kinase D2) which is involved in the phosphorilation of the class II histone deacetylase HDAC5 [34]. HDAC5 acts as a chromatin modifier and usually repress gene expression when not phosphorilated. It is thus possible that the mechanotransduction pathway involves a series of phosphorilation events leading to gene derepression through HDAC5 phosphorilation. It was shown that *klf2a* expression was absent in *prkd2* mutants which do not form valves. HDAC5 knock down is sufficient to rescue *klf2a* expression in the *prkd2* mutants, so it is possible that PrKD2 controls *klf2a* derepression through HDAC5 phosphorilation (Fig. 5a), which would constitute a key element of the mechanotransduction pathway. Further work will be needed to validate this hypothesis. The role of *klf2a* during valve development seems conserved in vertebrates as it is expressed in endocardial cells of the AVC in chick and mouse [26, 39], and because the knock out of *klf2* in mouse leads to valve defects [16]. Not surprisingly, it seems that other flow-dependent transcription factors are flow dependent during valve development. For example, the gene *egr1* has been shown to be expressed in a flow-dependent manner in the AVC of zebrafish [4]. Blood flow also activates expression of microRNA (miR), which in turn regulates the expression of gene targets and cell proliferation necessary for valve formation, such as *miR-21* [4]. Furthermore, other miR have been found to be activated by mechanical stress such as *miR-143* in the outflow tract, where another set of valves will develop. The impact of flow here is dramatic: it is certainly a set of mechanogenetic interactions as well as cross regulation through miR that explain the full set of cell response to flow observed in the developing heart.

While the flow activated signaling pathways and the gene response are becoming understood, the flow-sensing mechanism or mechanisms remain unclear. In vitro experiments suggest that different classes of stimuli are sensed by endothelial cells, such as flow direction, oscillation amplitude, and strain, yet the molecular mechanodetector remains undetermined in the embryo. Typical Ca2+-permeable non-selective cationic channels are top candidates for shear sensing in endothelial cells though their impact during heart development remains unstudied [40, 60].

### Early heart beat: comparison with engineered pumps

**Fig. 6:**
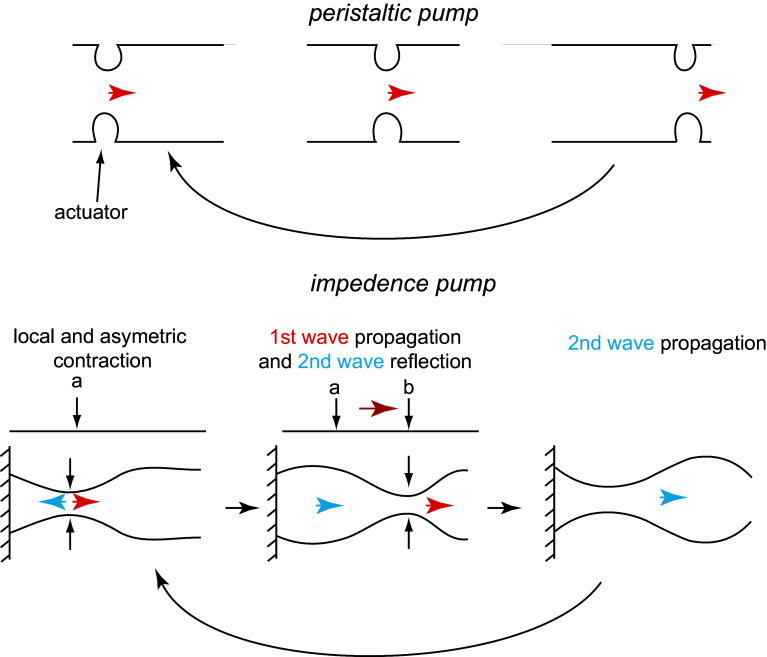
Schematic of the different mechanisms of a peristaltic pump (*top*) versus an impedance pump (*bottom*)

The remarkable pumping mechanism of the heart at its tube stage has been the subject of several investigations. What makes it so intriguing is that it works without valves and with the Reynolds and Womersley numbers less than unity (discussed in following sections), so non-linear inertial mechanisms are unavailable to generate unidirectional flows starting from cyclical stimulation. In a system so dominated by viscosity, only the dynamics of the flow boundaries can generate unidirectional flow. In the embryonic heart, this is achieved by a specific peristaltic contractile wave, which also forms the basis of engineered peristaltic pumps. In these pumps, an actuator occludes a local region of a fluid-filled elastic tube. As this actuator moves along the tube, it forces the fluid in the tube to move in the same direction (Fig. 6).

Despite these superficial similarities, Forouhar et al. [19] identified several discrepancies between the dynamics of a peristaltic pump and the embryonic cardiac wall. Live imaging of the zebrafish heart at 30 hpf suggests that the active contraction is localized in the atrial precursor and that elastic waves propagate passively both upstream and downstream from the contraction region. This contrasts with the unidirectional motion of peristaltic pumps. Moreover, they observed a non-linear dependence of the flow rate on the heart beat frequency, which would not be present for the standard peristaltic pump. Similarly, they estimated a phase shift between pressure and flow rate. Based on these observations, they concluded that the embryonic heart is an impedance pump rather than a peristaltic pump (Fig. 6). The working mechanisms of the heart as an impedance pump is counterintuitive, and investigations are ongoing. Gharib and collaborators employed both experimental and computational models to identify and quantify potential mechanisms [3, 30]. In these models, unidirectional flow is obtained by pinching an off center region of a relatively flexible tube attached to more rigid tubes. The resulting waves are reflected at the interface with the more rigid tubes (Fig. 6). The superposition of these waves provides the local high pressure required to pump the flow. Excitations at frequencies near the natural frequency of the system maximize the efficiency. Furthermore, Loumes et al. [45] showed that a multilayer wall, composed of an outer thin layer and a thicker elastic inner layer representing the cardiac jelly, significantly increases the efficiency, suggesting a non-trivial mechanical role of the cardiac jelly. Theoretical and experimental models of impedance pumps and their relation to the embryonic heart are reviewed, for example, by Miller [63] and Männer et al. [47]. All these efforts indicate that the embryonic cardiac function is more complex than a simple peristaltic pump. However, the actual pumping mechanisms remain unresolved. As already pointed out by [63], impedance pump mechanisms have been tested for a large range of Reynolds (≈10–100) and Womersley (≈10–48) numbers, but not at the same flow conditions as in the the embryonic heart. In the embryo, the Reynolds and Womersley number is smaller than one (at least at the discussed stages). Under this flow condition, we show in the next session that traveling pulse waves (like in the passive adult human aorta) should not be expected. Consistently, Männer et al. [47] discuss experiments suggesting that the passive wave described by Forouhar et al. [19] might actually be the result of a coordinated active contraction of the cardiac wall along the entire length of the heart. Thus, questions remain regarding the full workings of the embryonic heart pump.

### Clinical relevance of deficient heart biomechanics

It is obvious that abnormal heart growth and development is deleterious for heart function leading to diseases. Since blood flow and mechanotransduction are essential for the control of cardiovascular development, improved interventions might depend upon better understanding of the molecular and mechanical roots of congenital diseases affecting heart development. For example, infants born with a hypotrabeculated ventricle exhibit compromised hemodynamics; conversely, hypertrabeculated ventricles can lead to an impaired diastolic function that can ultimately impede the filling of the ventricle [7, 72]. Valve defects are also associated with flow sensing, and there is accumulating evidence that the regulatory mechanisms governing normal valve development and flow also contribute to human valve pathology [2]. In humans, cardiac valve defects account for 20–30 % of all congenital cardiovascular malformations, with an incidence rate of ≈5 % in live births [31]. It is also anticipated that mutations affecting heart function, including cardiomyopathies [61], will also affect the underlying endocardium and the vascular network function and development. Overall the anatomy of the vascular network and of the heart is highly variable among vertebrates, which in turn broadens the range of forces and related stimuli that endothelial cells experience. However, the signaling pathways and mechanotransduction cascade are anticipated to be strongly conserved. Further work will be necessary to clarify this issue and identify clinical opportunities.

## Control mechanisms of blood flow in the developing vasculature

The mechanisms that regulate blood flow in the developing vasculature are just being discovered, though they are recognized as essential given the role of hemodynamic stimuli in mediating development. Both the mechanical and anatomical properties of blood vessels contribute to regulate blood flow in the vascular network. In this context, we consider three factors that are fundamental: the capacitive behavior of the dorsal aorta, the evolving topology of the developing vascular network, and the viscous and cellular character of blood. Here we discuss how these factors affect mechanisms that control blood flow during development, emphasizing how these mechanisms differ between the embryonic and the mature vasculature.

### Vessel wall elasticity: the viscous Windkessel effect

As the contraction of the heart is pulsatile, the flow in the vessels proximal to the heart also presents a strong pulsatile behavior. However, moving away from the heart, the flow is somehow rectified such that this pulsatility is significantly reduced, and the blood flow does not cease between systolic contractions of the ventricle as it does at the aortic root. In the adult vasculature, this rectification mechanism is associated with the elasticity of the vasculature and is regulated by a mechanism known as the Windkessel effect [62]. During systole, blood pumped rapidly from the heart generates the pulse wave, which inflates the aorta. During diastole, the elastic restoring force of the arterial wall propels the blood volume stored in the aorta into the rest of the vasculature. Each branching junction in the arterial tree reflects and transmits the pulse wave, the cumulative effect of which is that it provides an approximately frequency-independent impedance and rectification of the pulsatility at the aortic root into a relatively steady mean flow, deep in the vascular tree [42].

**Fig. 7:**
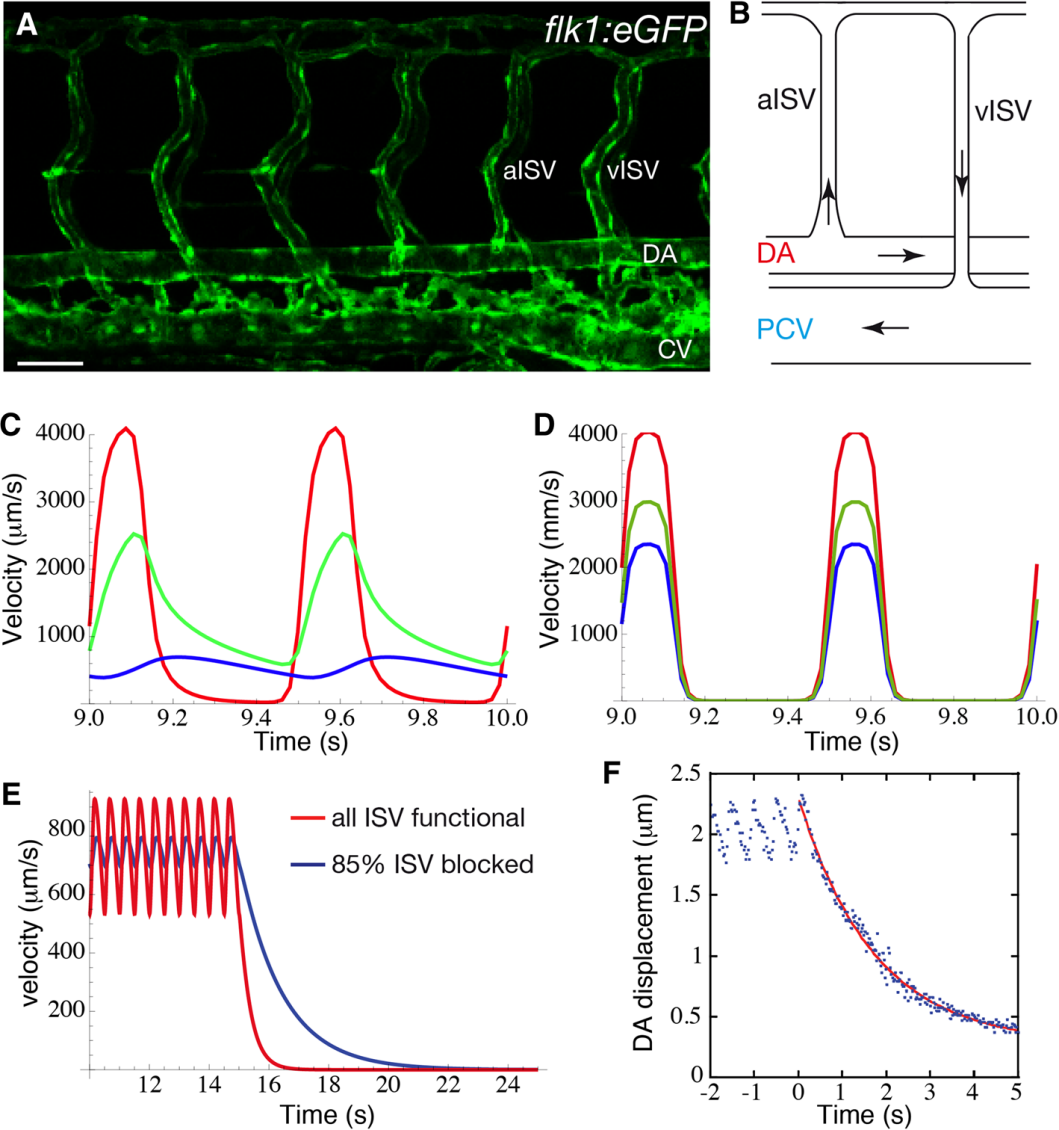
**a** Visualization of the vascular network shape in the trunk of a *Tg(flk1:eGFP)* and **b** a schematic of one of the vascular loops formed by arterial (aISV) and venous (vISV) intersegmental vessels (ISV). **c**, **d** Red blood cell velocity in the dorsal aorta (DA; *red*), in an ISV (*green*), and in the posterior caudal vein (PCV; *blue*), for an elastic (**c**) and a rigid (**d**) model of the vasculature. **e** Decay of the velocity in the DA following a stop in heart contraction computed for a control (*blue*) and for a mutant where 85 % of the a/vISV are blocked (zero diameter; increased hydraulic resistance). **f** Movement of the DA ventral wall following the stop in heart contraction ($t=0$; *blue dots*), and exponential fitting of the experimental data (*red line*). Adapted with permission from Anton et al. [1]

There is a similar rectification of the pulse in the embryo, reflected in the relative pulsatility of flow in the dorsal aorta (DA), the intersegmental vessels (ISV), and the posterior caudal vein (PCV) of the zebrafish at 72 hpf [1]. The anatomy of the zebrafish caudal vasculature is relatively simple at this stage, with the flow in loops formed by intersegmental artery-vein pairs connecting the DA and the PCV (Fig. 7a, b). Anton et al. [1] observed a progressive reduction in the pulsatility of the flow profile, with a 57 % reduction of the pulsatility amplitude in the PCV, where the flow appears almost steady, compared to the DA. Moreover, the pulse in the DA and in the ISVs is out of phase (Fig. 7c). If the heart is stopped, the velocity of the red blood cells (RBCs) decays to zero in about 5 s rather than instantaneously as would be the case for rigid vessels (Fig. 7e). Indeed, cyclic expansion of the DA with the same frequency as the heart beat is observed, and when the heart is stopped, the DA wall displacement is also observed to relax over about 5 s (Fig. 7f). These results suggest that the DA acts as a capacitor, resembling the behavior of the adult aorta, though without a true pulse wave or finite inertia [1].

**Fig. 8:**
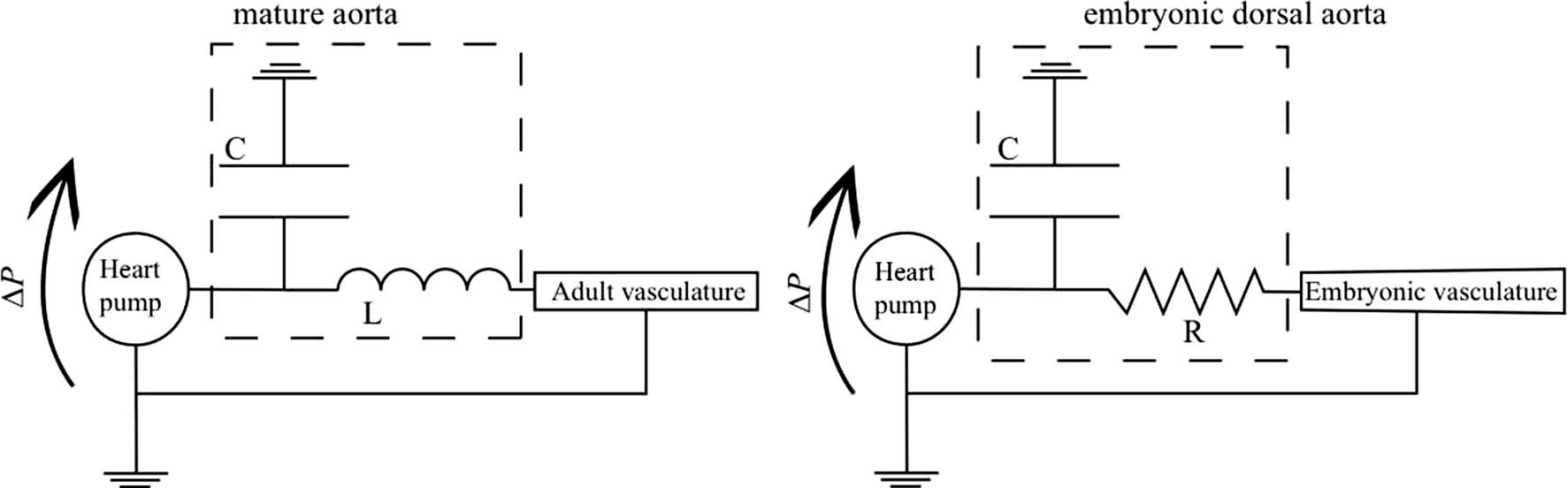
Electric analog illustrating the different mechanisms regulating the Windkessel effect in the mature (*left*) and embryonic aorta (*right*). In both systems, the elasticity of the wall confers a capacitive behavior of the vessel. However, the pressure difference $\Delta P$ (analogous to applied voltage) across the capacitive component of the aorta is mainly balanced by an inductance *L* representing the inertia of the fluid. In contrast, the capacitive component of the dorsal aorta is balanced by a resistance *R* (damping element), representing the viscous resistance of the embryo

The viscous analog of the Windkessel effect that has been recently proposed to rectify the pulse in the vasculature of the developing embryo is different in many ways to the adult Windkessel [1]. The flow regimes, and therefore the physical mechanisms regulating this capacitor behavior, are qualitatively different in the adult compared to the embryo. This difference in flow regime is expressed quantitatively by the Womersley number ($W\!o = D\sqrt{\rho \pi f/4\mu }$, vessel diameter $D$, pulse frequency $f$, and blood viscosity $\mu$), which quantifies the ratio between the time expected for the flow profiles to adapt due to viscous effect relative to time scales of the unsteady forcing of the heart beat. In the adult aorta, $W\!o \ge 10$, which is large. This means that the flow profiles are dominated by inertia rather than viscous effects. The result is flatter profiles, as opposed to the rounded approximately parabolic profiles of viscosity dominated flow, and the flow velocity and the pressure can be out of phase. In contrast, $W\!o \approx 0.01$ in the DA of the embryo, so the viscous forces cause velocities to adapt almost instantaneously to changes in pressure, and their profiles across vessels will be approximately parabolic. The hemodynamics are linear in the viscous limit, so the entire circulation would share a common phase, precisely reflecting the heart beat, if the blood vessels were rigid (Fig. 7d). The out-of-phase system and the suppression of the pulse require vessel elasticity. Using an electric circuit analogy, a rigid vessel model in the viscous limit would be entirely resistive, whereas the wall deformations would play a role analogous to a capacitor (by comparison, the blood inertia in the adult circulation would correspondingly be modeled as an impedance). From this perspective, the embryonic DA is conceptually like a capacitor in parallel with a resistor (Fig. 8).

The relative importance of viscosity versus inertia in the embryo when compared to the adult is reflected by the mathematical form of the equations that govern flow in the circulatory network. In the adult, the pressure obeys a wave equation (mathematically hyperbolic), and the pulse travels as a wave along the vessel with a speed that is inversely related to the distensibility of the wall. The aorta is inflated by the pressure pulse wave [42]. In contrast, the pressure in the embryo is governed by an equation analogous to that which would govern diffusive heat transport (mathematically parabolic), reflecting the diffusive character of viscous flow. A mechanical model [1] of the zebrafish network based on the heat-like equations reproduces the main features of the flow in the various segments (Fig. 7c). Interestingly, this same model also predicts that the elasticity of the DA significantly decreases the work required by the heart. In other words, it is relatively easier to inflate the DA in systole than to push blood through all the vessels, as would be required if they were rigid.

These differences between the adult versus the embryo imply differences in how observations of circulatory flow and deformation of the vessel walls can be interpreted. In the adult, the speed of the pressure wave depends on the vessel compliance. Therefore, the observed wave speed provides estimates of the mechanical properties of, say, the aorta, providing means of diagnosing pathological changes (e.g., hardening). The same is not true for the embryo, where changes in mechanical properties due to diseases or mutations are rather reflected in changes in the relaxation time scale. There is no pulse wave per se in the embryo. An estimate of this time scale is deduced from RBC motion and its cessation after the heart is stopped (Fig. 7e). The stiffer the vessels, the shorter the relaxation time will be. We point out, however, that the relaxation time constant does not just depend on the local elasticity, but rather on the balance against the hydraulic resistance of the entire system [1]. The larger the hydraulic resistance, the longer the relaxation time will be. The hydraulic resistance depends on both the geometry of vessels (strongly on their diameters: ${\propto} 1/D^4$) and on the effective blood viscosity, which depends on the hematocrit. Thus, it is not only a *local* measure of any property that is required in interpreting relaxation times of mutants with different hematocrit and/or vascular anatomy.

### Vascular topology regulates blood perfusion and vascular remodeling

The perfusion rate of the different parts of the developing vascular network is regulated by the topology of the vascular network. The regulation mechanisms share similarities with the mature microvasculature, but differ significantly in larger vessels, especially at bifurcations because of the different flow regimes. These are characterized by the Reynolds number (Re), which represents the ratio between the inertia and the viscous forces and in that way is analogous to the Womersley number $W\!o$ but for the steady component of the flow. In the adult vessels, there is a large range of Re: it is Re ≈ 10^2^–10^3^ in large arteries and Re ≪ 1 in capillaries. In the embryo, the diameter of *all* vessels is similar to the capillaries of the adult, so Re ≪ 1, and flow is dominated by viscous forces. Flows in this regime are typically called *Stokesian*, accurately governed by equations that do not include inertia factors. This flow regime has several implications on the way the vasculature geometry controls blood flow in development.

Stokesian flows are well known to be insensitive to geometric details, such as the curvature of the vessels, small details about the cross-sectional area, or the specific shape of a junction between vessels. Vortical structures develop in the adult aortic arch due to inertia, but not at low Re. Instead, the flow in curved vessels behaves almost as in straight vessels, and the relation between the pressure and the flow rate (the hydraulic resistance) is well approximated by the Poiseuille law, which is only accurate for larger Re if the vessels are perfectly straight (and Re ≤ 2300). In the embryo, there is none of the flow separation, recirculation, or sustained swirl that might exist at higher Re. Another specific example of geometric insensitivity involves the angle between two vessels at a bifurcation: this is not an important hemodynamic parameter at the low Re numbers of embryonic systems. This contrasts observations at higher Re, where such features can enhance, suppress, or even reverse shear stress. The shear stresses associated with this Stokesian regime have been proposed to have a role in the looping of the tubular heart [70]. It is worth notice that the initial stage of heart looping occurs properly in the absence of heart contraction and can happen after microdissection of the linear heart tube in zebrafish [54].

Viscosity also causes a rapid ‘forgetting’ of the flow history as conditions change. The adjustment to a new geometry (e.g., from the heart into the aorta) is rapid; there is essentially no *entrance length* for Re ≪ 1. In contrast, for the adult aorta, the entrance region is many diameters long, and the velocity profile is sensitive to the initial conditions for the entire length of the largest arteries. In the embryo, the inertial forces are so low that the flow profiles adapt within about one diameter of the entrance to the geometry of the vessel, and from there assume an approximately unchanging flow profile along its entire length.

**Fig. 9:**
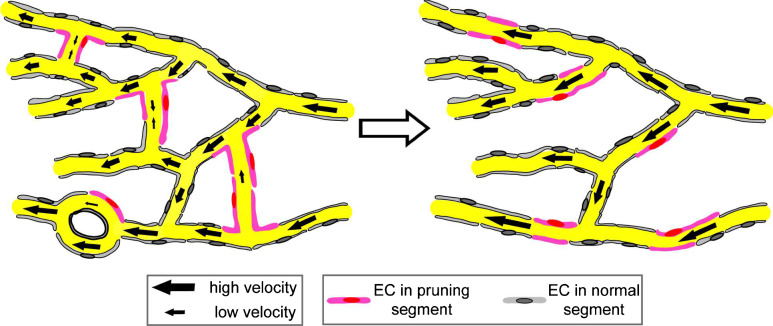
In the primitive vasculature (*left*), endocardial cells located at vessel segments (*pink*) that exhibit low and unstable blood flow undergo lateral migration, leading to vessel pruning. This process simplifies vasculature with reduced numbers of internal vessel loop (*right*). Adapted from Chen et al. [13]

A principal consequence of these different geometric insensitivities is that resistance to blood perfusion depends principally on the cross-sectional area of the vessels, specifically the fourth power of the diameter *D* ^4^ (if they are modeled as exactly round), and is linearly proportional to their length *L*. The position of the bifurcations is important, though not their geometric details. A different combination of these parameters can change the flow rate significantly in single vessels, and thereby contributes to define unique mechanical cues controlling vascular development. Capillaries with reduced flow rates were found to correlate with pruning in the developing brain vasculature [13]. Early in development, the capillary network of the middle brain is complex and characterized by many internal loops and redundant branches. Superfluous and redundant branches can have low shear stress, which seems to stimulate their removal and the subsequent remodeling of the network in a way that reduces its complexity [13, Fig. 9]. Overall, the complete loss of blood flow leads to impaired pruning but not absence of pruning [36]. Thus, it seems that the cells respond to subtle blood flow changes between neighboring blood vessels in determining if a given blood vessel will prune. It will be interesting to understand how endothelial cells sense and communicate these flow differences during the pruning process. Such processes also potentially modulate development in other ways, such as in branching morphogenesis and by setting arterial versus venous identity [11, 51, 53].

### Self-regulation of blood flow by red blood cells dynamics

The mechanisms presented in the previous sections do not necessarily depend fundamentally on the cellular character of blood. Under many conditions, neglecting these effects is justified because blood can behave as a homogeneous Newtonian fluid with viscosity several times that of the plasma due to the suspended blood cells, mostly RBCs. With this approximation, the pressure and flow rate in a vessel are coupled via the linear Poiseuille law: $U_{\text{mean}} \propto \Delta \rm{P D}^2$. However, when flowing in vessels of similar size to the blood cells (~10 μm), the cellular character of blood can alter its effective viscosity [59]. It is well known that apparent viscosity of blood depends on the diameter of the vessel [59], on the rigidity of the RBCs [22], and on the hematocrit [59]. Without data, it often must be assumed that the effective viscosity of blood is uniform in the whole embryo. However, the hematocrit, in particular, varies considerably in the embryo, which might affect the flow and the stresses it induces on the endothelium. Hematocrit changes arise because of the topology of the network [22] and the onset of haematopoiesis. Obrist et al. [55] considered a model for the dynamics of RBCs in a simple capillary network. He assumed simply that when a RBC arrives at a bifurcation, it flows into the vessel that provides the larger pressure gradient. The effective viscosity in each capillary segment was set accordingly to the instantaneous local hematocrit. This phenomenological model predicts that the RBCs distribute irregularly even in a nearly uniform network. A steady condition is never observed even if the imposed flow rate is steady. In some capillaries the concentration of the RBCs is much higher than in others. Some capillaries can even be occluded by the RBCs, preventing the capillary being perfused. Therefore, RBC dynamics can potentially contribute to the remodeling process as proposed by [13] (Fig. 9), where pruning follows flow reduction. This phenomenon was suggested to regulate the micro-circulation of the adult brain, but it can also be expected to play a similar role in the embryo, where the flow regime is similar.

**Fig. 10:**
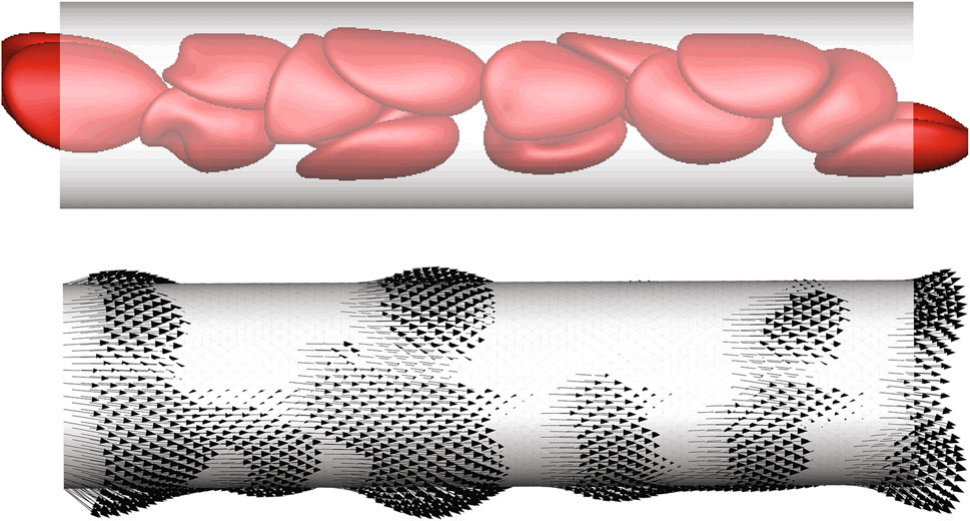
Simulations results for red blood cell traction footprint on a vessel wall [20]. The *top panel* shows the RBC position at a particular time; the *lower panel* shows the corresponding instantaneous tractions exerted on the wall. The vessel diameter is *D* = 12 μm; the shear rate is *U*/*D* = 119 s^−1^, with *U* the mean flow velocity in the capillary. Adapted from [20]

Even in a steady blood flow, its cellular character will induce stress fluctuations associated with the passing of individual cells. Freund and Vermot [20] recently computed the instantaneous flow forces on endothelial cells using a detailed flow simulation model with explicit representation of realistically deformable RBCs. These simulations showed that the wall shear stress associated with a passing RBC can lead to peak values of the flow stresses many times larger than the time average. This stress “footprint” on the endothelial cells thus includes a high-frequency component (Fig. 10), which might itself constitute a mechanotrigger. Considering that the hematocrit is very low when blood flow starts and increases to $Ht\approx 0.3$ (in small vessels), we can expect that not only the mean wall shear stress (WSS) but also its dynamic profile and the underling frequency spectrum change significantly during development. This provides potential mechanical cues specific for each developmental stage. However, future detailed characterization of the WSS in the developing embryo will be needed to support any role of this mechanism in vascular development.

### Mechanotransduction and mechanogenetics during angiogenesis

Endothelial mechanotransduction and its role in angiogenesis is well documented. The recent discovery of piezochannels and advances in live imaging have now shown that the Piezo1 (Fam38a) channels functions as a sensor of shear stress [40, 60], determining vascular structure, and that primary cilia are involved in low flow sensing during early angiogenesis. Cilia response is mediated by transient receptor channels such as Trpp2 (PKD2, PC2) in vivo in zebrafish [25] and in mouse endothelial cell culture [40, 50]. Both Piezo 1- and Trpp2-mediated endothelial cell response to flow is characterized by an increase in calcium levels (Fig. 5b).

Further downstream, flow leads to an increase in NO production and gene expression such as *klf2a, cxcr4, s1p1, hath6* [18], *alk1*, and *miR-126* and *vegf* (via *klf2a*) [52]. Overall blood flow is known to stabilize the vascular network by activating cell adhesion and stopping cell migration [58], but it can also favor angiogenesis early both in veinous [25] and arterial vessels [40, 52] depending on the specific forces endothelial cells sense and the type of vessels they will eventually form (Fig. 5c). Thus, the mechanical environment and chemical factors couple to modulate the endothelial cell behavior necessary to form and maintain the appropriate blood vessel type.

## Summary and outlook

Blood flow forces play a fundamental role during angiogenesis and valve development in the heart, and are potential regulators of vascular remodeling over the different developmental stages. While the cellular response to flow, and the biology of mechanotransduction has been extensively reviewed [21, 38], descriptions of fluid dynamic mechanisms regulating blood flow and flow forces at the early stages of development have received less attention. Yet these flow force mechanisms are key features of development, particularly in their capacity to modulate some developmental changes in the vascular system. Thus, we have provided an overview on the biofluid dynamics of blood flow during early developmental stages. Our discussion has focused on four major points: (a) the pumping mechanisms of the heart, (b) the elastic capacitive behavior of the blood vessels, (c) the functional topology of the vessels, and (d) the cellular character of blood.

The pumping mechanism of the heart evolves during development, transitioning from a valveless tubular heart, which induces unidirectional flow by traveling contraction waves, to a looped heart, which generates unidirectional flow via the active motion of the so-called cushions and then later by the valve leaflets. Still, many opportunities remain for understanding the mechanical characteristics of the developing heart. Analogies with engineered pumps do not fully correspond to the working principles of the embryonic heart, and many proposed physical mechanisms which might be important in adult animals do not apply in the low Reynolds and Womersley numbers of the embryo. Thus, a necessary step will be to provide a theory for the pumping mechanism of the developing heart. This will be facilitated by the fast imaging techniques enabled by light sheet technology which can track the intricate dynamics of the heart [46, 48]. Understanding how the heart works will help understand the phenotype of mutations at a laboratory level, and eventually aid diagnosis of prenatal pathological conditions at a clinical level.

Although the heart is the major actor of the cardiovascular system, it is essential to recon that it is fundamentally coupled to the vascular network. The mechanical and topological properties of the network are essential in regulating blood perfusion and flow forces in the different regions of the cardiovascular system. Specifically, we have discussed how the elasticity of the vessel walls provides a capacitive behavior to the DA that reduces the pulsatility of the flow in the peripheral vessels, therefore differentiating the mechanical cues of the arterial and venous sides of the system. These phenomena have been purely investigated in the embryo, and have the potential to inspire new comparative mechanotransduction studies in different regions of the developing cardiovascular system. Moreover, we have summarized the role of several topological features of the vessels—vessel radius and curvature, bifurcations, and network organization—in the regulation of perfusion. Despite its importance in the angiogenic process, there are few reported studies of this self-regulation of the embryonic vessel network. However, the more-studied adult capillary networks share the low Reynolds numbers of the embryonic vasculature and are thus a potentially useful starting point for investigations of angiogenesis. Identifying similarities and differences between the largest embryonic vessels and also the low-Reynolds-number microvasculature of the adult is important for understanding the potential utility of the embryo as a model for important adult processes. Insights into the flow responsive embryonic angiogenesis could help, for example, to better understand the biomechanics and mechanogenetics leading to the abnormal vascular networks of tumors [37].

Similarly, the explicit role of RBCs (and other blood cells) in mechanotransduction and flow regulation during development has not been extensively considered, despite the evidence that the unsteady character of fluid forces can indeed trigger developmental changes. With simulation models, we have discussed how the presence of RBCs is expected to lead to highly fluctuating shear stress on the endothelium, how RBCs may cluster in and plug specific “redundant” vessels of the vascular network, and how these represent potential self-regulation mechanisms of angiogenesis and blood perfusion. Future investigations are required to provide in vivo evidence of these potential mechanisms.
